# RAB7 GTPases as coordinators of plant endomembrane traffic

**DOI:** 10.3389/fpls.2023.1240973

**Published:** 2023-08-17

**Authors:** Cecilia Rodriguez-Furlan, Rita Borna, Oliver Betz

**Affiliations:** School of Biological Sciences, Washington State University, Pullman, WA, United States

**Keywords:** Rab7, RabG, endosomes, vacuole, tethering, stress, GTPases

## Abstract

The ras gene from rat brain (RAB) family of small GTPases is highly conserved among eukaryotes and regulates endomembrane trafficking pathways. RAB7, in particular, has been linked to various processes involved in regulating endocytic and autophagic pathways. Plants have several copies of RAB7 proteins that reflect the intricacy of their endomembrane transport systems. RAB7 activity regulates different pathways of endomembrane trafficking in plants: (1) endocytic traffic to the vacuole; (2) biosynthetic traffic to the vacuole; and (3) recycling from the late endosome to the secretory pathway. During certain developmental and stress related processes another pathway becomes activated (4) autophagic trafficking towards the vacuole that is also regulated by RAB7. RAB7s carry out these functions by interacting with various effector proteins. Current research reveals many unexplored RAB7 functions in connection with stress responses. Thus, this review describes a comprehensive summary of current knowledge of plant RAB7’s functions, discusses unresolved challenges, and recommends prospective future research directions.

## 1 Introduction

Ras gene from rat brain (RAB) GTPases are a class of regulatory proteins essential for eukaryotic cell endomembrane trafficking (Touchot et al., 1987; Homma et al., 2021). RABs are found on different cellular membranes and function as molecular switches cycling between an active GTP-bound state and an inactive GDP-bound state. RABs assist transport between cellular compartments by controlling various endomembrane trafficking processes, including vesicle generation, mobility, tethering, and fusion. Overall, RABs are essential for maintaining the proper organization and function of the endomembrane systems in eukaryotic cells.

RAB GTPases exhibit a high degree of conservation across eukaryotic organisms, with twenty-three RAB subfamilies identified across species (Elias et al., 2012). The RAB plant sequences group in only eight clades encompassing six subfamilies common to both yeasts and mammals, which are RAB1, RAB5, RAB6, RAB7, RAB8, and RAB11 while three, RAB2, RAB18, and RAB22, are exclusively present in plants and mammals and are not detected in yeasts. Therefore, using the 57 Arabidopsis encoded RAB genomic sequences, another nomenclature was proposed for the Arabidopsis subclasses RABs (A-H). Based on their sequence homology, the Arabidopsis categories are related to the original RAB subfamilies as follows: RABA=RAB11 and RAB25, RABB=RAB2, RABC=RAB18, RABD=RAB1, RABE=RAB8 and RAB10, RABF=RAB5 and RAB22, and RABG=RAB7 and H=RAB6 (Teh and Moore, 2007).

In Arabidopsis, RABA have been related the regulation of the late steps of secretion, RABB and RABD to the endoplasmic reticulum-Golgi transport, RABE to the regulation of vesicle secretion and early endocytosis, and RABF and RABG regulate traffic to the vacuoles. The RAB7 cluster in plants (RABG in Arabidopsis) has arisen as a subject of considerable attention in the scientific community due to its potential to significantly enhance plant resilience in the face of biotic and abiotic stress (Tripathy et al., 2021). Numerous studies across diverse plant species have consistently demonstrated that the overexpression of RAB7 proteins leads to a remarkable improvement in plant performance when subjected to various stress conditions. This phenomenon has stimulated a growing body of research into the underlying mechanisms through which RAB7 proteins benefit stress tolerance. This review will provide specific examples of how RAB7 affects various cellular activities, from functions common to animals and yeast to plant-specific functions. We will review our current knowledge of RAB7’s impact on plant growth and stress responses. Finally, we will highlight the open questions and potential future research directions to expand our understanding of RAB7’s function.

## 2 RAB7 functions as a molecular switch

**Figure 1:**
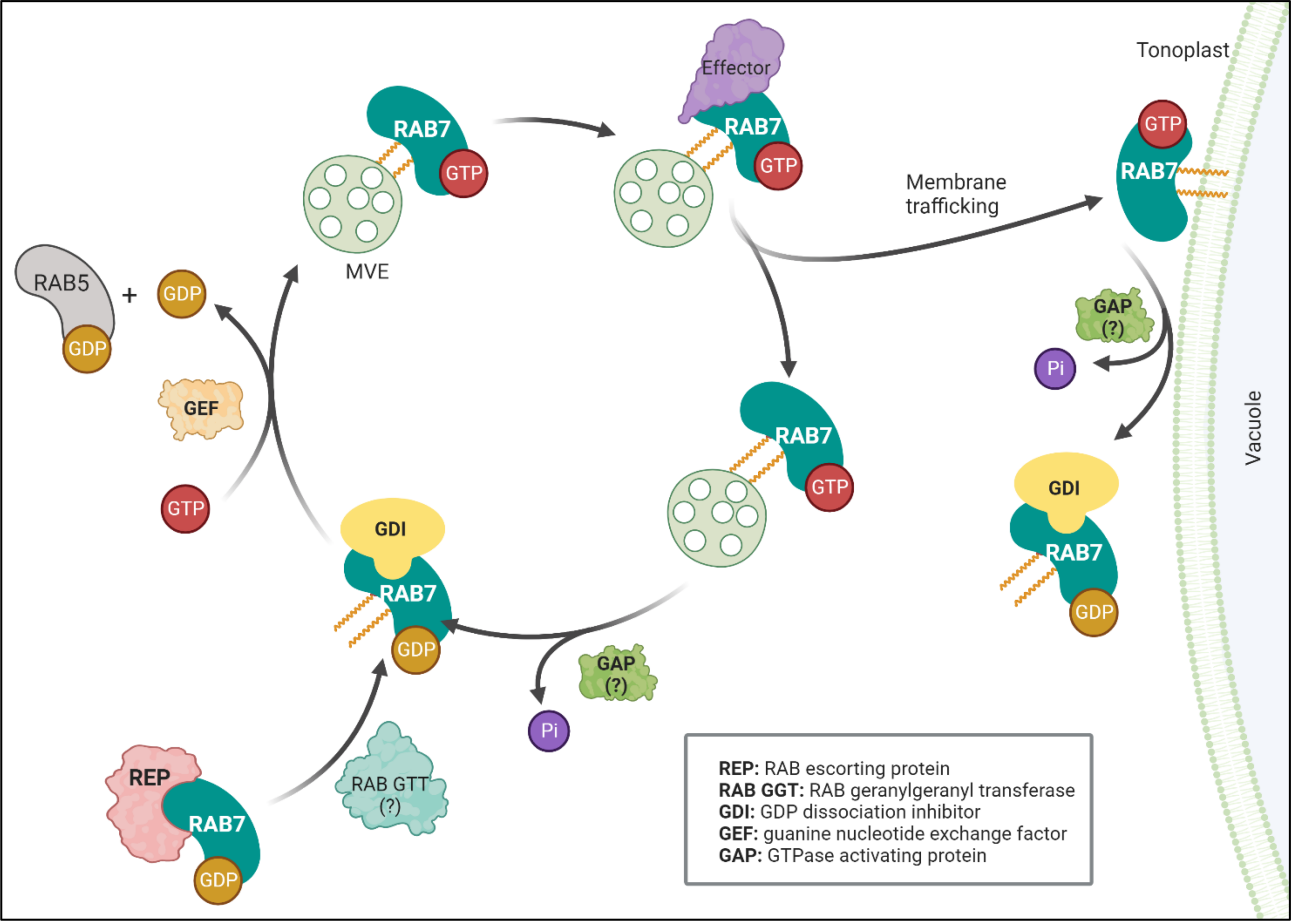
RAB7 functions as a molecular switch. RAB7 cycles between inactive (GDP-bound) and active (GTP-bound) states. A RAB escorting protein (REP) binds newly synthesized RAB7 associated to GDP and presents it to the geranylgeranyltransferase (RGT) and becomes prenylated. The prenylated protein can be solubilized in the cytosol by the GDP dissociation inhibitor (GDI), which protects the hydrophobic geranylgeranyl groups from the hydrophilic environment. A guanine nucleotide exchange factor (GEF) releases GDP and loads GTP to stabilize prenylated RAB7 in its active state to the MVE membrane. The GEF complex also acts as a GTPase activating protein (GAP) for RAB5 exchanging it for RAB7 at the maturating MVE. Once activated RAB7-GTP can interact with different effectors controlling membrane trafficking events like retrieval of proteins (recycling) or fusion with the vacuole (interaction with tethers). GTP hydrolysis by GTPase activating protein catalyzes RAB7 hydrolysis (GAPs interacting with RAB7 are still unidentified in plants, referred as: )?. Finally, a GDP dissociation inhibitor (GDI) releases the membrane bound RAB7 into the cytoplasm, reinitiating the cycle. Figure created with BioRender.com.

RAB7 is a member of the RAB family of GTPases, which switches between an active guanosine triphosphate (GTP)-bound state and an inactive guanosine diphosphate (GDP)-bound form (**Figure 1**). The proteins controlling the RAB switch are conserved among different organisms. They associate with membranes that are prenylated at their C-terminal cysteine residues by a RAB geranylgeranyltransferase (RGT). In *Arabidopsis*, heterodimers RGTA1-RGTB1 and RGTA1-RGTB2 can prenylate a wide range of RABs, including RABGs (Shi et al., 2016). The prenylated RAB7 can then be delivered to a target membrane, where a guanine nucleotide exchange factor (GEF) triggers GDP release and loading of GTP, thereby stabilizing RAB7 in its active conformation and allowing for specific effector proteins to be recruited to the membrane (Vetter and Wittinghofer, 2001). Eventually, RAB7 becomes inactivated by interacting with GTPase-activating proteins (GAPs), triggering GTP hydrolysis. The GDP-bound RAB7 is then released from the membrane into the cytoplasm by a GDP dissociation inhibitor (GDI), which solubilizes the prenylated tail until reactivation by GEFs (Zárský et al., 1997; Ueda et al., 1998).

## 3 The RAB7 group in plants

In animals, the protein RAB7 is involved in the late stages of endocytic trafficking, including the maturation of late endosomes and lysosomes and the degradation of cellular waste material through autophagy (Guerra and Bucci, 2016). In yeast, the RAB7 ortholog Ypt7p is essential for the maturation and fusion of late endosomes with the vacuole and for the homotypic fusion of vacuoles (Nordmann et al., 2012). Plants exhibit a higher copy number of RAB7 proteins when compared with other organisms, which may reflect the higher complexity of plant endomembrane trafficking. For example, there are five RAB7 homologs in rice: OsRAB7a1, OsRAB7a2, OsRAB7b1, OsRAB7b2, and OsRAB7b3 (Nahm et al., 2003); five RAB7 homologs in the moss *Physcomitrella patens* (Uemura and Ueda, 2014)*;* and eight RAB7 homologs have been described in the *Arabidopsis* genome: RABG1, RABG2, RABG3a, RABG3b, RABG3c, RABG3d, RABG3e, and RABG3f (Rutherford and Moore, 2002; Vernoud et al., 2003).

Studying individual functional contributions using knockout genetic approaches is challenging due to the large size of the RABG family in *Arabidopsis*. Individual mutants did not show a significant phenotype; however, quadruple, quintuple, and sextuple mutants exhibited dwarfism in the early developmental stages. These mutants were fertile and eventually grew to a size similar to the wild type (Singh et al., 2014). The quintuple mutants of RABG3b, c, d, e, and f and the sextuple mutants of RABG3a, b, c, d, e, and f show deficits in biosynthetic and endocytic protein transport to the vacuole. Furthermore, these mutants have fragmented vacuoles, affecting lytic and storage vacuoles. The abnormalities displayed by several RABG3 isoform mutations suggest that RABGs play a vital role in vacuole trafficking and biogenesis. However, these processes should be essential for plant growth and development, resulting in severe phenotypes. The mild phenotypes observed in the RABG3 sextuple mutant could be attributed to the partial reduction in RABG3f expression levels (the insertional mutant is not a null allele, instead it is a knockdown) and the remaining expression of the other family members RABG1 and RABG2.

To better assess the function of individual members, several publications used instead point mutations of the GTPase active site, to generate constitutively active or inactive RABG3 forms. RAB proteins feature two “switch regions” that accommodate the gamma phosphate of GTP, which causes significant conformational changes between their inactive and active states. Mutating S/T into N in the switch-I motif GXXXGK(S/T) disturbs the coordination of the gamma phosphate, thereby lowering GTP affinity (Gabe Lee et al., 2009). This GDP-locked RAB7 sequesters GDI and GEF proteins, acting as a dominant negative (DN). The DXXGQ motif in the switch-II region Q catalyzes GTP hydrolysis. The Q to L mutations impede GTP hydrolysis, making the protein GTP-locked and constitutively activated (CA) RAB7.

The overexpression of DN-RABG3c inhibits the vacuolar targeting of soluble and membrane proteins (Bottanelli et al., 2012). Additionally, CA-RABG3f overexpression results in enlarged pre-vacuolar compartments known as multivesicular bodies or endosomes (MVBs, MVEs), modified vacuole morphology, hindered protein vacuolar trafficking, and finally, affected whole plant development (Cui et al., 2014). Moreover, inducible DN-RABG3f overexpression showed vacuole protein traffic defects leading to inhibition of root development in a dexamethasone dose-dependent manner, co-related with a dose-dependent increase in expression of the DN protein. Therefore, in animals, yeast, and in plants RAB7s are central regulators of late endosome fusion with the lytic compartment. Additional functions regulating homotypic vacuole fusion and autophagy regulation are conserved. However, plant-specific functions like regulation of traffic to the specialized lytic and storage vacuoles has also been described (Cui et al., 2014; Singh et al., 2014).

## 4 RAB7’s role in vesicle maturation during traffic to the vacuole

In plants, the trans-Golgi network (TGN) sorts biosynthetic cargo but also receives and sort materials internalized from the plasma membrane by endocytosis (acting as an early endosome) (Rosquete et al., 2018). The TGN cargo can be sorted into compartments that will mature by developing intraluminal vesicles and receive the name multivesicular endosomes (MVEs), also known as multivesicular bodies, late endosomes or pre-vacuolar compartments, to finally fuse with the vacuole membrane, i.e., the tonoplast. As soon as the MVEs leave the TGN, can be recognized by the presence at their membranes of the protein RAB5 (Ebine et al., 2014). In *Arabidopsis*, the RAB5 GTPase, RABF2b (also known as ARA7), bounds the membranes leaving the TGN until the complex MON1 (SAND1)-CZZ1AB recruits the RAB7/RABG3f (Cui et al., 2014; Singh et al., 2014).. The MON1 (SAND1)-CZZ1AB complex functions as a GEF activating RABG3f and as a GAP for RAB5 that is then released from the MVEs (**Figure 1**). Accordingly, DN-RABG3f has been detected to be associated with the MON1, CZZ1A, and B complexes (Rodriguez-Furlan et al., 2019). A proportion of DN-RABG3f is associated with membranes and restricted to MVEs, indicating that RABG3f activation is necessary for its arrival to the tonoplast (Cui et al., 2014). Similarly, in rice, it was shown that only when b2oth MON1 and CZZ1 are present, they can interact with OsRAB7b3, while MON1 by itself can interact with OsRAB5a (Pan et al., 2021). The available information suggests a model where MON1 recognizes RAB5 proteins while recruiting CZZ1 to act as a GAP for RAB5 while recruiting and activating RAB7 (**Figure 1**).

## 5 RAB7 association with the tethering complex HOPS

**Figure 2:**
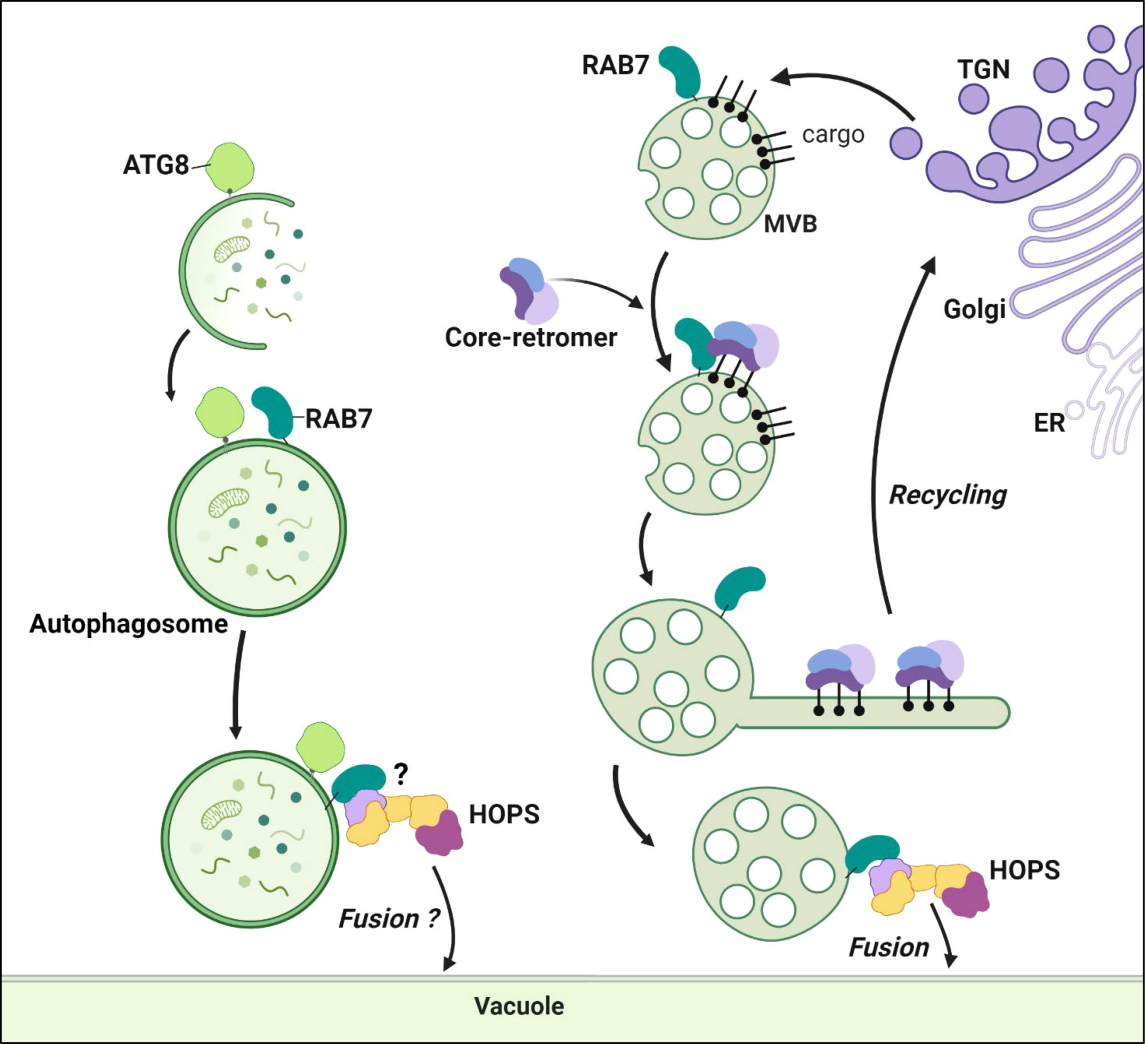
RAB7 at MVEs and autophagosomes. RAB7 anchors the core-retromer to the MVEs membrane. The core-retromer is formed by VPS35, VPS26 and VPS29 (depicted with three different colors in the figure). At the MVEs the retromer interacts with cargo proteins sorting them for recycling back to the TGN. In mammalians the retromer forms extensions or tubules rescuing the proteins from degradation, such structures have not been described in plants yet. RAB7 also interacts with the HOPS complex at the MVEs favoring the fusion of the vesicles with the tonoplast, releasing the contents in the vacuole lumen. RAB7 is co-localizing and co-immunoprecipitating with ATG8. However, its function at autophagosomes remains uncharacterized. The HOPS complex role facilitating fusion of autophagosomes with the tonoplast also remains to be studied (referred as )?. Figure created with BioRender.com.

Once activated, RAB7 plays a crucial role in membrane fusion by interacting with tethering complexes that facilitate the initial contact between membranes. RAB7 interacts with the homotypic fusion and protein sorting (HOPS) complex formed by the vacuolar protein sorting (VPS) subunits VPS11, VPS16, VPS18, VPS33, VPS39, and VPS41 (Balderhaar and Ungermann, 2013). Then, the fusion events are made possible by the presence of soluble N-ethylmaleimide-sensitive factor attachment receptor (SNARE) proteins on both membranes (Lipka et al., 2007). Q-SNAREs (such as syntaxins of plants, SYP) are present on the target membrane, and R-SNAREs (like vesicle-associated membrane proteins, VAMP) are observed on the vesicle. The Q- and R-SNAREs associate to enable fusion events. In *Arabidopsis*, the HOPS complex interacts with the Q-SNARE SYP22 and the R-SNARE VAMP713, allowing MVE-tonoplast fusion (Takemoto et al., 2018) (**Figure 2**).

VPS39 in yeast attaches to RAB7 (Ypt7) in endosomes. At the same time, VPS41 binds to a Ypt7 located at the tonoplast connecting both membranes (Lürick et al., 2017). In *Arabidopsis*, only partial information shows that VPS3, VPS33, VPS39 and VPS41 can co-immunoprecipitate with RABG3f (Takemoto et al., 2018; Rodriguez-Furlan et al., 2019). Similar to yeast, VPS41 and RAB7 (RABG3f or RABG3c) colocalize only in the tonoplast (Hao et al., 2016; Brillada et al., 2018; Jiang et al., 2022). Furthermore, VPS33 interacts directly with the Q-SNARE SYP22, probably recognizing the SNARE (Brillada et al., 2018). Therefore, the recruitment and architecture of the RAB7-HOPS-SNARE complex in plants still need to be fully elucidated.

Beyond its role in MVE-tonoplast fusion, the RABG3-HOPS complex interaction is required for vacuole formation and homotypic vacuole fusion, as evidenced by the HOPS and RABG3 mutants’ fragmented vacuole phenotypes (Singh et al., 2014; Hao et al., 2016; Brillada et al., 2018). In this line of evidence, RABG3f and VPS39 can be detected colocalizing at contact points between adjacent vacuoles.

The HOPS complex was detected in autophagosomes, along with the autophagy-related protein ATG14, a phosphatidylinositol-3-phosphate kinase (Wang et al., 2022). ATG14 is co-immunoprecipitated and colocalized with RABG3a and RABG3f. Consistent with these findings, when DN-RABG3f or DN-RABG3a is overexpressed, cells accumulate autophagosomes in the cytoplasm (Lürick et al., 2017). Therefore, it is possible that RAB7 proteins observed in autophagosomes can be involved in HOPS complex assembly, thereby facilitating autophagosome fusion with the tonoplast (**Figure 2**). However, such a hypothesis still needs to be tested.

## 6 RAB7’s association with the core retromer complex

RAB7 can interact with another complex, the core-retromer VPS35-VPS26-VPS29 (**Figure 2**). The VPS35 subunit interacts with RABG3f to anchor the cytosolic VPS35-VPS26-VPS29 complex to the MVEs (Zelazny et al., 2013). In *Arabidopsis*, the core-retromer has extra copies of a few subunits, including VPS35A, VPS35B, VPS35C, VPS26A, VPS26B and VPS26C; however, there is only one copy for VPS29. Interestingly, the activation of RABG3f promotes the VPS35A-RABG3f interaction, as evidenced by the greater co-immunoprecipitation of VPS35A with CA-RABG3f compared to DN-RABG3f (Gabe Lee et al., 2009). The inhibition of the chemical interaction between RABG3f and VPS35A interrupted the trafficking of endocytic and synthetic cargo toward the vacuoles (Gabe Lee et al., 2009). Similar phenotypes are observed in mutants of the different subunits of VPS26, VPS29, and VPS35 (Nodzyński et al., 2013; Munch et al., 2015; Jha et al., 2018). Triple mutant *vps35a1b2c1*, double mutant *vps26a1b1*, and single mutant *vps29* all exhibited deleterious developmental phenotypes, highlighting the biological relevance of the different subunits (Yamazaki et al., 2008). However, VPS35A and C redundantly regulate vacuolar protein sorting, impacting PVC morphology, while VPS35B’s role seems dispensable in these pathways (Munch et al., 2015). Therefore, multiple subcomplexes likely exist since all VPS35 isoforms interact with VPS26 and VPS29 (Zelazny et al., 2013). This interaction might be tissue- or process-specific, depending on the needs of the particular cell.

The core retromer is implicated in recycling vacuolar sorting receptors from the MVE to the TGN (Hu et al., 2022). The ALIX protein associates with the VPS26-VPS29 dimer in the cytosol, thereby stabilizing the interaction and recruiting VPS35. ALIX mutations show a defect in protein delivery to the vacuole and miss the localization of vacuolar sorting receptors. Therefore, a model where ALIX interaction precedes VPS35-RABG3f interaction is proposed. In this updated model, ALIX recruits VPS26-VPS29, and the three proteins then interact with VPS35, which is anchored to the MVEs by RABG3f. VPS35 can then interact with and recognize the vacuolar sorting receptors at the MVEs and recycle them back to the TGN (**Figure 2**).

Overall, the retromer-RAB7 interaction has particularities unique to plant systems. The identity and mechanistic of retromer cargo retrieval is still debated and is an interesting research topic in plant cells (Heucken and Ivanov, 2018).

## 7 RAB7 and plant cell death regulation during developmental processes

In plants, programmed cell death (PCD) plays a significant role in diverse growth, developmental processes. PCD can be triggered by different factors during development, including differentiation induction and age-related senescence. For example, differentiation-induced PCD is the final step in the maturation of specific cell types like the xylem tracheary elements, root cap, or anther tapetum layer (Plackett et al., 2011; Bollhöner et al., 2012; Fendrych et al., 2014; Olvera-Carrillo et al., 2015). Meanwhile, age-induced PCD occurs during the senescence of organs or the entire plant at the end of its life cycle (Thomas, 2013).

The role of RABG3b in regulating PCD has been hypothesized in the context of xylem-tracheary element differentiation (Ménard et al., 2015). CA-RABG3b overexpression was discovered to induce autophagy, as evidenced by the formation of autophagic structures in the cytoplasm of developing tracheary element cells (Kwon et al., 2010; Kwon et al., 2011). An increase in autophagy is linked to the disintegration of cellular contents and organelles, followed by vacuole collapse, releasing hydrolytic enzymes into the cytosol for total cellular breakdown (Hara-Nishimura and Hatsugai, 2011). In cells with deficient autophagy, such as *atg5-1* mutants, cells expressing DN-RABG3b, or cells with RNA interference-mediated RABG3b knockdown (RABG3bRNAi), vacuole collapse is either delayed or absent (Kwon et al., 2010). Before vacuole collapse, events such as increased vacuole size, transport and activation of vacuolar lytic enzymes, acidification of vacuoles, and the degradation of the material delivered by autophagy are observed (Van Doorn et al., 2011). These processes are linked to RABG3 activity, which implies that it may play a role in controlling the pathways leading to programmed cell death (Kwon et al., 2010).

## 8 RAB7 and plant cell death regulation during pathogen attack

PCD is also involved in stress, and immune responses. Upon pathogen detection the plant’s immune system often induces a controlled PCD process usually called the hypersensitive response (HR). The term “hypersensitivity” arises from the unusually fast and extended cell death around the pathogen site of infection to prevent its spread (YIN et al., 2022). Interestingly, when exposed to PCD inducers, such as fungal toxin fumonisin B1 and the bacterial pathogens *Pst DC3000* (*AvrRpm1*) and *Pst DC3000* (*AvrRpt2*), CA-RABG3b overexpressing plants accumulated a large number of autophagic structures and displayed accelerated and expanded cell death (Kwon et al., 2010). Additionally, VPS35B retromer mutants are defective in autophagic degradation and immunity-associated PCD when exposed to *Pst DC3000 (AvrRpm1) and Pst DC3000 (AvrRpt2)* (Munch et al., 2015). Therefore, it is tempting to propose that RABG3b and retromer function positively contribute to immunity-associated PCD by activating autophagic cell death and containing the spread of the infection. Supporting this notion, knocking down the RAB7 (TaRAB7) gene expression in *Triticum aestivum* resulted in a higher abundance of the fungus *Puccinia striiformis f.* sp. *tritici* on infected leaves compared to control leaves, which suggests a role for TaRAB7 in controlling infection (Liu et al., 2012).

Another theory proposes a link between the enhanced autophagy that precedes PCD and the spatial confinement of cell death, thereby avoiding necrotic cell death and the spread of toxic signals that could induce PCD in nearby cells (Patel and Dinesh-Kumar, 2008; Escamez et al., 2016). This hypothesis explains the observed enhanced senescence and prolonged cell death upon overexpression of CA-RABG3b (Kwon et al., 2010; Kwon et al., 2013). However, further investigation is necessary to elucidate the precise role of RAB7 in regulating plant cell death.

## 9 Are RAB7 MVEs turned into exosomes during biotic interactions?

Numerous microorganisms that engage in symbiotic or pathogenic interactions with plants exhibit specialized cellular structures that invade host cells and remain enveloped by membranes derived from the host (Ivanov et al., 2010). The same applies to *Phytophthora infestans*, whose hyphae penetrate plant cells and remain surrounded by an extrahaustorial membrane that interfaces the host and the pathogen. RAB7 (RABG3c) is found on the extrahaustorial membrane and is thought to participate in rerouting MVEs towards this membrane (Bozkurt et al., 2015). Another case is the *Rhizobium* bacteria that are individually internalized into symbiosome compartments. Symbiosomes are intracellular in nature but are considered apoplastic compartments separated by the plasma membrane (Ivanov et al., 2012; Coba de la Pena et al., 2018). As the symbiotic association develops, the symbiosome membrane also becomes labeled by RAB7 (Limpens et al., 2009).

Another case is the powdery mildew infection. Plant cells respond to the non-host pathogen’s presence by repolarizing their secretory pathway, producing a structure named papillae composed of cell walls and antimicrobial components, and depositing it around the fungal haustoria to halt fungal growth (Underwood and Somerville, 2008). The *mon-1* mutants in the *Arabidopsis* No-0 ecotype form defective papillae structures, leading to penetration and infection by the powdery mildew *Golovinomyces orontii (Go)* (Liao et al., 2023). The data implies that MON1 is essential for activating RAB7 during papillae formation. Accordingly, during papillae formation when cells are attacked by the fungus *Blumeria graminis f.* sp. *hordei (Bgh)*, RABG3c interacts with the component of the EXOCYST complex EXO70B2 located at the papilla membrane (Ortmannová et al., 2022). Therefore, it has been hypothesized that EXO70B2 may act by tethering RAB7 MVEs to the plasma membrane during papilla and encasement formation.

In each of these hypotheses, RAB7 MVEs are thought to reroute and fuse with the plasma membrane rather than the vacuole. However, this theory needs additional testing. During the developmental stage in which RAB7 is detected, VPS39 and VPS41 proteins are not present in the membrane of the symbiosomes (Gavrin et al., 2014). This suggests that the presence of RAB7 at these membranes serves a different functional role or that another fusion machinery (possibly the EXOCYST complex) is involved in tethering RAB7 MVEs with the plasma membrane.

## 10 RAB7 is highjacked by pathogens to infect cells

RAB7 appears to be important in defense against infection, which may explain why several pathogens target its activity to better penetrate or replicate in plant cells. In barley, the *Blumeria graminis f.* sp. *hordei (Bgh)* effector CSEP0162 protein interacts with the heat shock proteins of the plant and MON1, thereby directing them into aggresomes (Liao et al., 2023). The aggresomes are intracellular depositions of misfolded proteins turned into cytoplasmic inclusions (Verchot, 2011). These aggresomes are believed to be degraded by autophagosomes (Lamark and Johansen, 2012). Therefore, by hijacking MON1, CSEP0162 activity prevents the formation of the papillae encasements favoring the powdery mildew fungus infection in barley (Liao et al., 2023).

Positive-strand RNA viruses, such as tomato bushy stunt virus (TBSV) and carnation Italian ringspot virus (CIRV), exploit RAB7 activity to facilitate the formation of viral replication organelles (VROs) in plant cells (Feng et al., 2021a). VROs are membranous intracellular structures containing viral proteins and viral RNAs that sequester subverted host factors to facilitate replication and prevent cellular degradation. The depletion of RAB7 significantly inhibits TBSV and CIRV replication. The viral p33 replication protein interacts with RAB7, leading to the redistribution of RAB7 into the VROs. Deleting MON1 or CCZ1 impedes TBSV RNA replication, suggesting that activated RAB7 plays a proviral role. Furthermore, p33 was shown to interact directly with the retromer components VPS35, VPS29, and VPS26 to recruit them into VROs (Feng et al., 2021b). Therefore, it is proposed that the retargeting of RAB7 and the core-retromer into VROs by p33 enables the delivery of various retromer cargos, including lipid enzymes, all of which possess proviral functions. These findings indicate tombusviruses exploit RAB7 to redirect endocytic and recycling trafficking pathways to support efficient virus replication.

## 11 RAB7’s role in abiotic stress responses

Many research teams have overexpressed RAB7 in various plant species and have repeatedly observed an improvement in plant fitness in response to various abiotic stressful circumstances (Agarwal et al., 2007; George and Parida, 2011; Peng et al., 2014; Tripathy et al., 2017; El-Esawi and Alayafi, 2019). In *Arabidopsis*, overexpression of the RABG3e gene increases tolerance to osmotic and salt stress, decreases the formation of reactive oxygen species, and shows improved recovery from osmotic stress, thereby increasing stress response efficiency (Mazel et al., 2004). Similarly, RAB7 overexpression in rice enhanced the responses to salt stress by enlarging the vacuolar size in both the roots and the leaves, suggesting increased sodium sequestration as a stress response mechanism (Peng et al., 2014; Tripathy et al., 2017). In addition, these plants retained photosynthetic activity and grana integrity, which enabled proper chloroplast function under salt stress. In a different study, RAB7 overexpression in rice boosted water retention, growth rate, and oxidative stress responses to heat and drought (El-Esawi and Alayafi, 2019). These findings potentially indicate a role for RAB7 in sodium sequestration, cellular homeostasis maintenance, and ROS reduction to enable normal cellular function under abiotic stress conditions. Additionally, during ammonium stress roots of czza1b1 mutants show arrest of growth and accumulation of autophagosomes in the cytoplasm, consistent with a role of RAB7 regulating autophagy during stress (Robert et al., 2021). However, further research is required to dissect the mechanism behind the RAB7-orchestrated response.

## 12 RAB7 more than just tethering: future topics of exploration

Recent advances in our understanding of RAB7-mediated endomembrane traffic are summarized in this review, emphasizing the diverse regulatory mechanisms that have evolved in land plants. The primary takeaway from this analysis is that there is still much to learn about RAB7 roles. This includes but is not limited to further characterization of the RAB7 compartments, understanding the biochemical interactions at play, and determining how RAB7 function contributes to the outcomes seen in mutant and overexpressing plants. Several promising directions for future study are suggested, as follows:

RAB7 has been detected in late MVEs (Cui et al., 2014) and autophagosomes (Kwon et al., 2013) and is even associated with TRAPPIII (Kalde et al., 2019), which is a complex detected in the TGN. The function of RAB7 in autophagosomes and potentially in TGN is still unknown. Further analyzing RAB7 interactions in different organelles will help us understand its function.

RAB7 it is in vesicles that likely fuse to the plasma membrane during a pathogen attack. However, the nature and contents of those compartments remain a matter of discussion. The mechanism by which such compartments are redirected and fused with the plasma membrane remains to be fully described.

RAB7-CA-overexpressing plants accumulate ubiquitinated proteins, autophagosomes, and MVEs and exhibit extensive cell death. An avenue of research could be analyzing the RAB7 contribution to containing PCD by facilitating the degradation of proteins and protein aggregates that can be toxic for cells.

RAB7 functions intricately connected to phosphatidylinositol 3-phosphate (PI3P), a vital membrane marker that is present in MVEs and autophagic compartments (Noack and Jaillais, 2017). In plants, PI3P synthesis occurs through the activity of the class III phosphatidylinositol 3-kinase (PI3K) complex, composed of the VPS34 kinase, ATG6, VPS15, and either VPS38 or ATG14 as the fourth subunit. Notably, an interaction between RAB7/RABG3a from *N. benthamiana* and the subunits ATG14 and VPS38 has been observed *in vitro* (Wang et al., 2022). However, the precise mechanisms underlying the association between PI3P and RAB7 in regulating autophagic traffic remain unresolved, representing another interesting research topic.

Creating conditional or inducible RAB7 mutants could help understand its specific role during abiotic stress responses in different plant species.

We are especially optimistic about the recent developments in proteomics, including proximity labeling technologies (Kim et al., 2019) that could give us spatial resolution (Han et al., 2018) and enough data to start answering many of these questions. Finally, we are particularly interested in examining the similarities and differences across various plant species. The fact that overexpressing RAB7 in numerous plant species widely improves their fitness under stress demonstrates the similarities shared by organisms with vastly different physiologies and evolutionary histories. Thus, improving our understanding of these critical pathways across the plant kingdom will pave the way to a complete picture of how the plant endomembrane system is regulated.

## Author contributions

CR-F: conceptualized, wrote the entire review, prepared the figures. RB contributed to the writing process of four sections of the manuscript. OB contributed to the abiotic stress section writing. All authors contributed to the article and approved the submitted version.
